# The relationship between dietary patterns derived from inflammation and cognitive impairment in patients undergoing hemodialysis

**DOI:** 10.3389/fnut.2023.1218592

**Published:** 2023-08-03

**Authors:** Yan Zhuang, Xinmei Wang, Xuanrui Zhang, Qian Fang, Xinyi Zhang, Yan Song

**Affiliations:** ^1^Medical School (School of Nursing), Nantong University, Nantong, Jiangsu, China; ^2^Blood Purification Center, Affiliated Hospital of Nantong University, Nantong, Jiangsu, China

**Keywords:** dietary pattern, cognitive impairment, inflammation, hemodialysis, end-stage kidney disease

## Abstract

**Introduction:**

Dietary patterns were shown to be closely related to inflammation, which was independently associated with cognitive impairment (CI) in patients undergoing hemodialysis (HD). However, it remains unclear the influence of dietary patterns derived from inflammation on CI in this population. This study aimed to examine the association between dietary patterns derived from C-reactive protein (CRP) and interleukin-6 (IL-6) and CI in patients undergoing HD.

**Methods:**

Dietary intake was obtained from the simplified quantitative food frequency questionnaire. Reduced rank regression (RRR) was used to extract two dietary patterns, with IL-6 and CRP as response variables. Cognitive function was examined by the Montreal Cognitive Assessment (Beijing version). Venous blood was drawn for measuring IL-6 and CRP levels. Multivariable logistic regression was used to investigate the association between dietary patterns and CI.

**Results:**

Dietary pattern derived from IL-6 was not significantly associated with CI. The third quartile of dietary pattern, which used CRP as the response variable, significantly contributed to the increased risk of CI (AOR 8.62, 95% CI 1.47–50.67) after controlling age, sex, education level, marital status, and residential pattern (*p*-for-trend = 0.028). After considering hypertension and diabetes, physical activity level, anxiety and depression, smoking and drinking status, social support, energy intake, and the dietary pattern derived from IL-6 (*p*-for-trend = 0.026), the relationship between the dietary pattern derived from CRP and CI remained significant (AOR 14.54, 95% CI 1.40–151.13).

**Conclusion:**

Dietary pattern associated with high CRP level, including high intake of rice, liquor, fruit, tea and coffee and low intake of dark vegetables and juice, contributed to the increased risk of CI. The association between the consumption of seafood, sweet beverages, and alcohol and CI is yet to be established. However, they may be dietary contributing factors to inflammation in patients undergoing HD.

## Introduction

Hemodialysis (HD) is the most widely available form of maintaining life for patients with end-stage kidney disease. Approximately 2.62 million people worldwide depend on HD treatment for survival (1). Cognitive impairment (CI) has been reported to be prevalent in 87% of individuals receiving HD globally (2). CI seriously affects the prognosis, hospitalization, and complications, thus decreasing patients' long-term quality of life and increasing the burden on families and society (3). Notably, the mortality rate of patients undergoing HD with CI was 1.7–2.5 times higher than that of patients without CI (4). However, the influencing factors that cause CI remained unclear (5). Increasing evidence has shown that diet is potentially associated with cognitive function in this population group (6, 7). The Mediterranean diet (MD), which mainly includes vegetables, fruits, seafood, beans, and nuts, has shown a promising association with a slow rate of cognitive decline and a reduced risk of CI (8). A randomized controlled trial (RCT) that included 447 participants has shown that MD was associated with the improvement of cognitive function (9). A Longitudinal neuroimaging study has also reported that high adherence to MD was associated with a slow rate of β-amyloid, which was closely related to cognitive function (10). Additionally, a prospective study that involved 3,831 participants over 65 years and followed up for 11 years has suggested that the Dietary Approaches to Stop Hypertension (DASH) dietary pattern was correlated with a reduced risk of cognitive function decline (11).

Low-grade chronic inflammation is common in patients undergoing HD. With the decline in renal clearance rate, the levels of circulating proinflammatory cytokines increase gradually (12). Oxidative and carbonyl stresses promoted by the uremic environment are highly proinflammatory (13). The microbiological quality and impurities in the dialysate may also lead to inflammation (14). In addition, a significant association between diet and inflammation has been presented in previous studies as well (15). A 5-year, single-blind, multicenter, controlled feeding trial randomized 66 participants into three groups: MD plus extra virgin olive oil, MD plus nuts and a low-fat diet (LFD). Compared with LFD, both MD groups had lower levels of interleukin-6 (IL-6) (16). In a meta-analysis of RCTs, DASH played an important role in decreasing the C-reactive protein (CRP) concentration (17). Plenty of studies have revealed that inflammation was a potential risk factor influencing cognitive function (18–20). A systematic review including 170 studies has revealed that patients in the CI group had higher levels of IL-6, compared to the control group (21). In a cohort study followed up for 6 years, higher levels of CRP, IL-1β, and fibrinogen were correlated with a higher risk of impaired attention (22). Therefore, dietary patterns leading to elevated inflammatory biomarkers may contribute to the development of CI. However, scientific evidence demonstrating the influence of dietary pattern related to inflammatory biomarkers on CI in patients undergoing HD is scarce. This study aimed to examine the association between dietary patterns derived from inflammatory biomarkers and cognitive impairment in patients undergoing HD.

## Methods

### Study design and participants

This cross-sectional study was conducted among patients undergoing HD using convenient sampling, with patient recruitment and data collection taking place between June and October 2022. All participants were recruited from a hemodialysis unit within a tertiary hospital in Nantong, in the Jiangsu province of China. Patients were eligible for the study if they were 18 years of age or older and undergoing HD for more than three months. Patients were excluded if they had psychiatric diseases, poor comprehension and hearing, and had abused alcohol and drugs for a long time. Patients who had taken antibiotics within 1 month or had severe inflammation within 6 months were also excluded. This study was reviewed and approved by the Institutional Review Board at Nantong University (2021-48), and all participants provided informed consent prior to participation.

The influencing factors of CI in patients undergoing HD were collected and analyzed based on the health ecology model in the study. According to previous publications, 15 influencing factors from five dimensions, including biological indicators, behavioral lifestyles, psychological factors, disease-related problems, and social environmental problems, were collected (23, 24). Following the calculation of sample size in the multifactor statistical analysis proposed by Kendall (25), the recommended sample size is usually 5–10 times the number of variables. A loss-to-follow-up rate of 20% has been considered, resulting in a required sample size between 90 and 180 patients. The collected influencing factors were included as covariates in the analysis to adjust for confounding effects on the relationship between dietary patterns derived from inflammatory biomarkers and cognitive impairment.

### Dietary intake

Dietary intake was assessed using a quantitative simplified food frequency questionnaire (FFQ). It has been reported that FFQ had great reliability (0.54–0.77) and validity (0.54–0.87) (26). The FFQ is divided into 25 food groups, such as rice, gruel, noodle and their products, dessert, fried foods, and so on. Participants were asked to choose the frequency of consumption for each food item from nine possible frequency responses, ranging from “never” to “three times a day”. In addition, the intake of juice, tea, and coffee was also collected. Face-to-face interviews were conducted with patients during their HD sessions. Researchers printed and completed the FFQ based on patients' responses. Standard portions of food were photographed using a palm-sized reference object to facilitate participants' understanding and accurately estimate their dietary intake. Patients who had unreliable daily energy intake (< 800 kcal/day or >4,000 kcal/day for men; < 500 kcal/day or >3,500 kcal/day for women) were excluded from the analysis.

### Cognitive function

Cognitive function was examined by the Montreal Cognitive Assessment Beijing version (MoCA-BJ), which is the most widely used screening tool to assess cognitive function. It has been reported that MoCA-BJ had high sensitivity (92.4%) and specificity (88.4%) in the identification of CI among the Chinese population (27). The MoCA-BJ includes eight cognitive fields: attention and concentration, executive function, memory, language, visuospatial construction, abstract thinking, computation, and orientation function. The total MoCA-BJ scores range from 0 to 30. The total scores would be increased by one point if participants were illiterate, primary, middle, or high school graduates (≤ 12 years of education). Participants with a score < 26 were defined as having CI (28). During dialysis sessions, executive and visuospatial construction sections in the questionnaire were filled out by patients themselves, and other sections were completed by researchers according to patients' responses.

### CRP and IL-6

A volume of 1.5 ml blood (fasting) was taken from the participants for measuring the concentration of IL-6 and high-sensitivity CRP using an enzyme-linked immunosorbent assay (ELISA) before hemodialysis. When the IL-6 concentration is >7 pg/ml or the concentration of CRP is >5 mg/ml, inflammatory responses are observed in the body.

### Demographics

Age, vintage, body mass index (BMI), and comorbidities, including hypertension and diabetes, were extracted from participants' medical records. Education level, drinking and smoking status, marital status, and residential pattern (living alone) were obtained from the demographic questionnaire. Education level was categorized as ≤ 12 years of education and >12 years of education. Marriage status was categorized as married, divorced, and unmarried.

### Questionnaire

All the questionnaires, which were each marked with a unique non-identifiable participant study code, were administered to the patients by the researcher during their dialysis sessions. The hospital anxiety and depression scale (HADS) and social support rate scale (SSRS) were used to examine patients' anxiety and depression and the characteristics of social support for patients, respectively. The General Practice Physical Activity Questionnaire (GPPAQ) is utilized for assessing physical activity (PA) levels and identifying those with “Inactive” PA. It was designed to take less than 1 min to complete and the patients can be categorized into one of four “Physical Activity Index” (PAI) categories, namely “Inactive”, “Moderately Inactive”, “Moderately Active”, and “Active” based on the results.

### Statistical analyses

Continuous variables that meet and do not meet the normal distribution were expressed as the mean ± standard deviation (SD), and median and interquartile range (IQR), respectively. Dichotomous variables are presented as numbers and percentages. An independent-sample t-test, one-way analysis of variance, and Mann-Whitney *U* test were performed to assess the distributions of general characteristics between CI and non-CI groups.

Reduced rank regression (RRR) analysis was used to extract two dietary patterns, with IL-6 and CRP as response variables, respectively. The RRR package was adopted to derive the dietary patterns by using the STATA statistical software version 16.0 (29). Factor loadings were analyzed to evaluate the association between 27 food groups and two dietary patterns. Food groups with factor loadings of ≥0.1 were positively correlated with the dietary pattern, while those with ≤ -0.1 were negatively correlated.

Logistic regression was used to examine the effect of dietary patterns on the risk of CI with odds ratios and 95% confidence intervals. The dietary pattern scores were calculated from the sum of the standardized intake of each food group multiplied by its factor loading, and the scores of dietary patterns were categorized into four groups based on the quartiles. The first quartile was used as the reference. In the multivariable-adjusted model, age, sex, education level, marital status, and residential pattern were considered in model 1, and model 2 was further adjusted for hypertension, diabetes, physical activity level, anxiety and depression, smoking and drinking status, social support scores, energy intake, and other dietary patterns. Moreover, the linear trend tests were conducted by incorporating the median value of four groups of dietary patterns as a continuous variable in the models. These statistical analyses were performed using the SPSS statistical software version 26.0. *P* < 0.05 were statistically significant.

## Results

**Figure 1:**
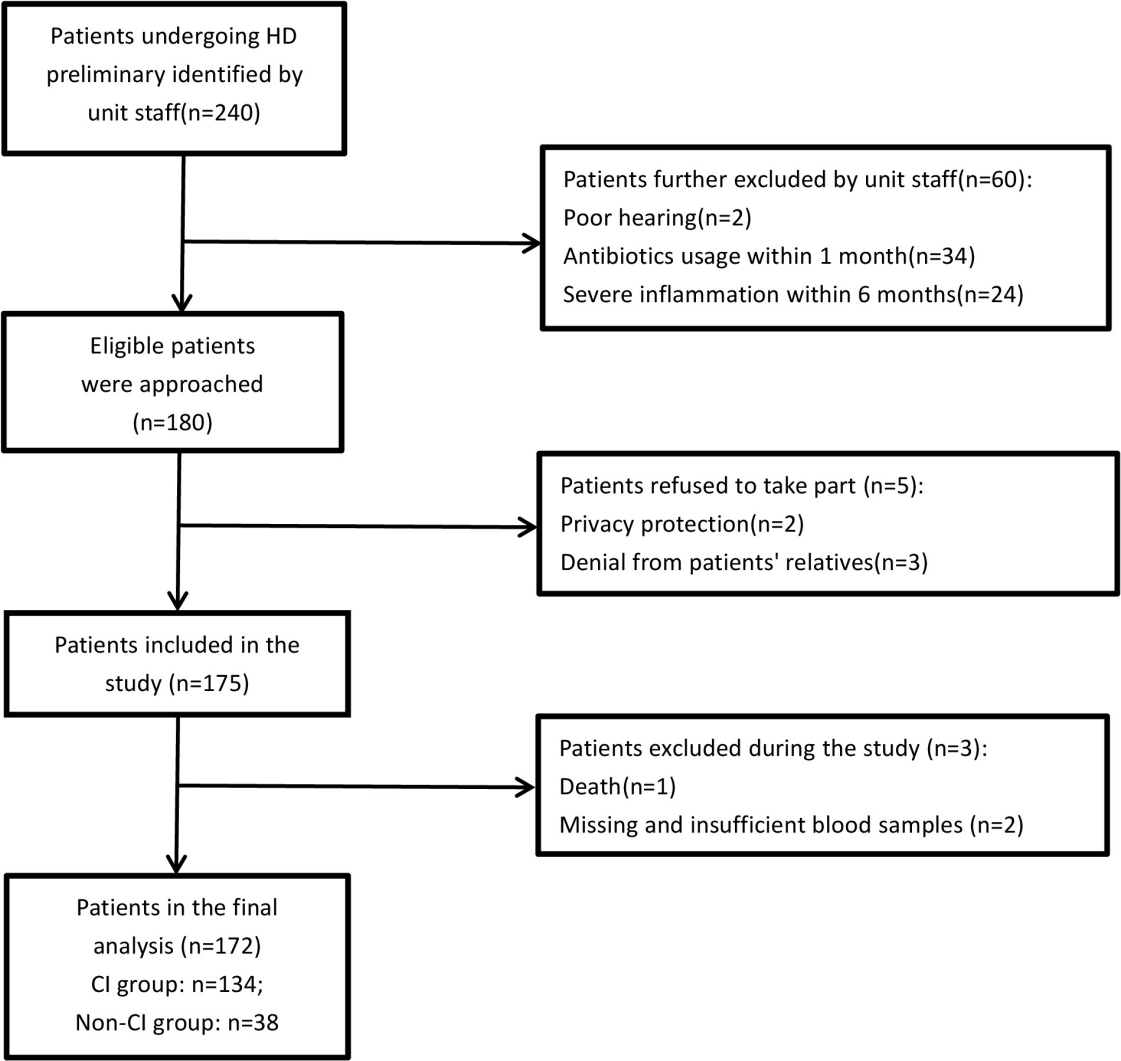
Flowchart of patients' recruitment. HD: hemodialysis; CI: cognitive impairment.

A total of 240 adult patients who had been undergoing HD for >3 months at the HD unit were preliminarily identified by unit staff. The unit staff further excluded patients who did not meet the eligible criteria and briefly explained the study to the potential patients during routine dialysis sessions. A total of 60 patients were excluded for the following reasons: poor hearing (*n* = 2), antibiotics usage within 1 month (*n* = 34), and severe infection within 6 months (*n* = 24). A total of 180 patients who expressed an interest in participating in the study were provided with the patient information sheets and discussed the study in more detail with the researchers. At least 48 h were given to the patients to decide whether to participate. If they were interested in the study after consideration, written consent was obtained from all participants prior to entering the study. A total of 175 patients ultimately participated in the study and signed the informed consent. Patients completed two separate interviews lasting about 20 min each and consisting of questionnaires and cognitive tests. During the study process, a patient who passed away during the study was excluded. Two patients were excluded due to missing or insufficient blood samples for analysis. The data of these three patients was completely missing at random, indicating that the probability of data being missed was entirely independent of its assumptions and other variables. Therefore, the study excluded the data and estimated any missing values from known variables. Finally, 172 patients were included in the analysis. A flowchart of included and excluded participants is shown in Figure 1.

A total of 134 (77.91%) participants whose score of MoCA-BJ lower than 26 were grouped into the CI group, and the remaining 38 (22.09%) participants entered the non-CI group. Among all the included participants, 40.12% were women and 59.88% were men. The comparison of general characteristics between CI and non-CI groups is shown in Table 1. There were significant differences in gender, age, education level, residential pattern, and marital status between the two groups. Compared to the non-CI group, the participants in the CI group tended to be older (42.24 ± 1.61 vs. 57.37 ± 0.98 years), fewer educated, and more women (18.42% vs. 46.26%).

**Table 1:** Comparison of general characteristics between CI and non-CI groups.

**Characteristics**		**MoCA-BJ**		***P-*value**
		**Non-CI (*n =* 38)**	**CI (*n =* 134)**	
Biological indicators	Women	7 (18.42)	62 (46.26)	**0.002** ^**a**^
	Age (years)	42.24 ± 1.61	57.37 ± 0.98	**< 0.001** ^**a**^
	Vintage (years)	2.00 (8.25)	4.50 (9.00)	0.39
	BMI (kg/m^2^)	22.29 (5.56)	21.99 (4.99)	0.82
	Education level			**< 0.001** ^**a**^
	• ≤ 12 years	21 (55.26)	116 (86.57)	
	•>12 years	17 (44.74)	18 (13.43)	
Social environmental factors	Marital status			
	•Married	27 (71.05)	128(95.52)	**< 0.001** ^**a**^
	•Divorce	1 (2.63)	4 (2.99)	
	•Never married	10 (26.32)	2 (1.49)	
	Residential pattern (living alone)	8 (21.05)	10 (7.46)	**0.034** ^**a**^
	Social support	40.11 ± 0.44	39.85 ± 0.49	0.87
Disease-related indicators	Hypertension	30 (78.94)	86 (64.18)	0.086
	Diabetes	4 (10.53)	30 (22.39)	0.11
Behavioral lifestyle	Physical activity			0.61
	•Inactive	2 (5.26)	4 (2.99)	
	•Moderately inactive	5 (13.16)	23 (17.16)	
	•Moderately active	12 (31.58)	32 (23.88)	
	•Active	19 (50.00)	75 (55.97)	
	Currently smoking	7 (18.42)	10 (7.46)	0.91
	Currently drinking	5 (13.16)	19 (14.18)	0.87
	Energy intake (kcal/kg/day)	24.32 ± 1.62	21.00 ± 0.66	0.06
Psychological factors	Anxiety/depression	2 (5.26)	5 (3.73)	0.68

^a^p < 0.05.

MoCA-BJ, Montreal Cognitive Assessment Beijing version; CI, cognitive impairment; BMI, body mass index.

RRR was used to extract two dietary patterns and explained 14.6% of the total variation of the response variables (IL-6 and CRP). Dietary pattern 1 (DP1) accounted for 8.2% of the variation of IL-6, and dietary pattern 2 (DP2) accounted for 6.4% of the variation of CRP. Factor loadings were used to describe the magnitudes and directions of the contributions of 27 food groups in two dietary patterns. In the DP1, food groups with factor loadings ≥0.1 were seafood, liquor, and sweet beverages, while those with factor loadings ≤ -0.1 were noodles, nuts, dumplings, and coarse cereals. As shown in Table 2, food groups with positive factor loadings greater than 0.1 included rice, fruit, tea and coffee, and liquor, while dark vegetables and juice had factor loadings < -0.1 in DP2.

**Table 2:** Factor loadings of food groups associated with dietary patterns 1, 2.

**Food/food groups**	**Dietary pattern 1**	**Dietary pattern 2**	
Rice	−0.028	0.215^‡^	
Gruel	−0.027	0.040	
Noodle and their products	−0.149^‡^	0.031	
Dessert	0.041	−0.037	
Fried foods	−0.030	−0.004	
Dumplings	−0.192^‡^	−0.090	
Coarse cereals	−0.121^‡^	−0.051	
Tuber	0.011	0.022	
Milk	−0.070	−0.018	
Eggs	0.090	−0.088	
Red meat	0.069	0.063	
Poultry	−0.001	−0.080	
Processed meat products	0.003	0.030	
Fish	−0.045	−0.056	
Seafood products	0.207^‡^	−0.082	
Bean products	−0.099	−0.034	
Nuts	−0.110^‡^	−0.071	
Dark vegetables	−0.031	−0.145^‡^	
Light vegetables	0.019	−0.029	
Mushrooms	0.064	−0.083	
Fruits	−0.058	0.193^‡^	
Sweet beverages	0.196^‡^	−0.039	
Beer	−0.061	−0.016	
Rice wine	−0.020	0.003	
Liquor	0.105^‡^	0.101^‡^	
Tea and coffee	0.062	0.104^‡^	
Juice	0.067	−0.145^‡^	
**Explained variation(%)**	**DP1**	**DP2**	**Total**
Food/food groups	3.1	3.2	6.3
Predictor	8.2	6.4	14.6

^‡^The absolute value of factor loadings >0.1.

DP1, dietary pattern 1 (dietary pattern derived from IL-6); DP2, dietary pattern 2 (dietary pattern derived from CRP).

The scores of dietary patterns were categorized into four groups based on the quartiles. General characteristics of patients undergoing HD in the first and fourth quartiles of two dietary patterns are presented in Table 3. Compared with patients in the first quartile, fewer patients in the fourth quartile of the DP1 were married. In the DP2, more patients in the fourth quartile were men, younger, current smokers, and lived alone. Meanwhile, fewer patients suffered from diabetes compared to those in the first quartile.

**Table 3:** Characteristics of patients in different quartiles of dietary patterns scores.

**Characteristics**		**DP1**			**DP2**		
		**Q1**	**Q4**	***P*-value**	**Q1**	**Q4**	***P*-value**
Biological indicators	Women	11 (22.58)	18 (41.86)	0.11	22 (51.16)	9 (20.93)	**0.004** ^**a**^
	Age (years)	55.6 ± 1.93	50.12 ± 2.06	0.06	57.42 ± 2.02	49.95 ± 1.73	**0.006** ^**a**^
	Vintage (years)	2.00 (8.50)	7.00 (9.50)	0.09	3.50 (7.80)	2.50 (11.00)	0.87
	BMI (kg/m^2^)	22.82 (5.60)	22.68 ± 0.57	0.60	22.01 ± 0.48	22.69 ± 0.48	0.32
	Education level			0.25			0.17
	• ≤ 12 years	21 (48.84)	28 (65.12)		33 (76.74)	35 (81.4)	
	•>12 years	22 (51.16)	15 (34.88)		10 (23.26)	8 (18.6)	
Social environmental factors	Marital status			**0.033** ^**a**^			0.18
	•Married	42 (97.67)	35 (81.40)		39 (90.70)	38 (88.37)	
	•Divorce	0(0.00)	2 (4.65)		0 (0.00)	3 (6.98)	
	•Never married	1 (2.33)	6 (13.95)		4 (9.30)	2 (4.65)	
	Residential pattern (living alone)	6 (13.95)	8 (18.60)	0.56	2 (4.65)	10 (23.26)	**0.01** ^a^
	Social support	41.79 ± 0.82	39.26 ± 1.18	0.08	39.91 ± 1.04	39.07 ± 1.08	0.58
Disease-related indicators	Hypertension	27 (62.79)	30 (69.77)	0.49	25 (58.14)	30 (69.77)	0.26
	Diabetes	7 (16.28)	5 (11.63)	0.53	11 (25.58)	4 (9.3)	**0.047** ^**a**^
Behavioral lifestyle	Physical activity			0.53			0.68
	•Inactive	1 (2.33)	2 (4.65)		1 (2.33)	2 (4.65)	
	•Moderately inactive	7 (16.28)	9 (20.93)		8 (18.60)	5 (11.63)	
	•Moderately active	9 (20.93)	13 (30.23)		11 (25.58)	9 (20.93)	
	•Active	26 (60.47)	19 (44.19)		23 (53.49)	27 (62.79)	
	Currently smoking	4 (9.34)	6 (13.95)	0.50	2 (4.65)	9 (20.93)	**0.02** ^**a**^
	Currently drinking	8 (18.60)	7 (16.28)	0.78	5 (11.63)	10 (23.26)	0.16
	Energy intake (kcal/kg)	24.53 ± 1.20	23.19 ± 1.45	0.48	20.11 (11.29)	23.87 (9.68)	0.19
Psychological factors	Anxiety/depression	3 (6.98)	0 (0.00)	0.24	1 (2.33)	1 (2.33)	1.00

^a^p < 0.05.

Q1, first quartile; Q4, fourth quartile; DP1, dietary pattern 1 (dietary pattern derived from IL-6); DP2, dietary pattern 2 (dietary pattern derived from CRP); BMI, body mass index.

Adjusted odds ratios (AOR) and the 95% confidence intervals for CI across the quartiles of each dietary pattern are presented in Table 4. DP1 was not significantly associated with the risk of CI. In model 1, the third quartile of DP2 significantly increased the risk of CI (AOR 8.62, 95% CI 1.47–50.67) after adjusting for age, sex, education level, marriage status, and residential pattern (*p*-for-trend = 0.028). Model 2 was further adjusted for hypertension and diabetes, physical activity level, anxiety and depression, smoking and drinking status, social support, energy intake, and dietary pattern derived from IL-6 (*p*-for-trend = 0.026), and it was found that the third quartile of DP2 significantly increased the CI risk compared to patients in the first quartile of DP2 (AOR 14.54, 95% CI 1.40–151.13).

**Table 4:** Logistic regression on the relationship between dietary pattern and cognitive impairment.

**Quartile of dietary pattern**	**DP1**		**DP2**	
	**Model 1**	**Model 2**	**Model 1**	**Model 2**
Q1	1.00	1.00	1.00	1.00
Q2	2.01 (0.40–10.04)	1.39 (0.14–13.71)	1.06 (0.24–4.72)	0.63 (0.15–6.20)
Q3	0.95 (0.22–4.08)	0.34 (0.41–2.75)	**8.62 (1.47–50.67)** ^**a**^	**14.54 (1.40–151.13)** ^**a**^
Q4	0.80 (0.21–3.07)	0.81 (0.14–4.77)	4.44 (0.85–23.29)	5.92 (0.61–57.11)
*P* for trend	0.64	0.55	**0.028** ^**a**^	**0.026** ^**a**^

^a^p < 0.05.

Q1, first quartile; Q2, second quartile; Q3, third quartile; Q4, fourth quartile; DP1, dietary pattern 1 (dietary pattern derived from IL-6); DP2, dietary pattern 2 (dietary pattern derived from CRP).

Model 1: Adjusted for sex, age, education level, marital status, and residential pattern; Model 2: Adjusted for hypertension and diabetes, physical activity level, anxiety and depression, history of smoking and drinking, social support, energy intake, and dietary pattern derived from IL-6.

## Discussion

The existing evidence from epidemiological, cross-sectional, and neuroimaging research has shown that diet plays a potential role in improving cognitive function. The significant association between diet and inflammation has been presented in previous studies as well. Additionally, as we know, inflammation is a potential risk factor influencing cognitive function. However, evidence examining the association between dietary patterns, inflammation, and cognitive function in patients undergoing HD is scarce. There were two main findings in this study. Two dietary patterns with IL-6 and CRP as response variables were identified to show the relationship between diet and inflammation. The dietary pattern that accounted for the variation in IL-6 was characterized by a high intake of seafood, sweet beverages, and liquor and a low intake of noodles, nuts, coarse cereals, and dumplings. The dietary pattern that explained the variation in CRP was characterized by a high intake of rice, tea and coffee, liquor, and fruit and a low intake of dark vegetables and juice. Additionally, the study showed that dietary patterns utilizing CRP as the response variable significantly contributed to the increased risk of CI, while dietary patterns derived from IL-6 did not.

CRP and IL-6 were used as the response variables to understand the dietary pattern in this study, which is in agreement with some previous studies aiming to examine the influence of diet on inflammation. The MONICA/KORA Augsburg cohort study extracted the dietary pattern with IL-6, IL-18, and CRP as the response in 981 middle-aged men (30), in which the dietary pattern related to inflammation was characterized by a high intake of meat, sweet beverages, and liquor. The lack of association between inflammation and meat consumption in the study may be attributed to the fact that patients undergoing HD were less inclined to consume meat due to concerns regarding high phosphorus intake (31). Different from the previous studies showing that long-chain n-3 polyunsaturated fatty acids in seafood decreased inflammation (32), our findings have shown that the intake of seafood increased the concentration of IL-6. The processing and storage of seafood may lead to an increase in inflammation due to the formation of dietary cholesterol oxidation products (COPs), which are heterogeneous compounds that occur during the processing and storage of cholesterol-rich seafood (33). COPs are linked to the induction of oxidative stress and subsequent inflammation (34). Similar to the previous study, sweet beverages were associated with an increasing level of inflammation in the study. Compared to rats fed with an *ad libitum* chow diet only, those supplemented with sweetened beverages exhibited elevated levels of tumor necrosis factor (TNF) and interleukin-1β (35). The sugar in sweet beverages resulted in a reduction of probiotics within the intestines and induced inflammation. Juice was found to be negatively correlated with inflammation in this study. It is consistent with several studies where researchers have observed a reduction in CRP level following juice consumption (36, 37). Juice is rich in anthocyanins, flavonoids, polyphenols, and vitamin C, which possess antioxidant and anti-inflammatory properties (38). Similar to our finding, high alcohol consumption was found to be positively correlated with elevated levels of CRP and IL-6 (39), although a study conducted on rat models demonstrated that alcohol could promote anti-inflammatory processes (40). A national health survey carried out in former West Germany suggested that non-drinkers and heavy drinkers had higher CRP concentrations than moderate drinkers (41). Alcohol consumption showed a *U*-shaped association with mean CRP values. Dosage may play an important role in the effect of liquor on inflammation.

A systematic review including 46 studies reported that vegetable-based and fruit-based dietary patterns were negatively associated with low-grade chronic inflammation (42). Similarly, in our study, dark vegetables were confirmed to be beneficial in reducing CRP concentrations. Vegetables have strong antioxidant and anti-inflammatory properties, which reduce the levels of inflammatory factors by inhibiting the production of free radicals (43). Furthermore, vegetables are rich in fiber that positively influences gut microbiota, which may contribute to the mitigation of inflammatory responses (44). However, the aforementioned findings on fruits were incongruous with our discovery that fruit consumption led to an elevation in CRP level. The potential reason for this discrepancy may lie in the patients' preference for high-sugar fruits, such as grapes and peaches, as reported by many participants. Low-sugar fruits, like oranges and kiwifruit, were often rejected by patients undergoing HD due to their high potassium content. It has been known that foods rich in sugar lead to an inflammatory reaction (45). The overconsumption of fructose in fruits or starch in rice provokes metabolic changes that result in low-grade chronic inflammation (46). Fructose consumption induces leptin resistance, resulting in elevated levels of leptin. However, excessive leptin levels can trigger adipocyte inflammation (47). In addition, leptin contributes to the release of reactive oxygen species and the recruitment of monocytes, which subsequently trigger an inflammatory response (48).

The study revealed that the consumption of tea and coffee was positively associated with inflammation. Results on the impact of tea and coffee on inflammation exhibit inconsistency. The presence of numerous polyphenolic compounds in tea and coffee is generally attributed to their anti-inflammatory properties (49). However, the processing technology of tea may affect its effect on inflammation. Fermented tea is commonly believed to be more effective in reducing lipids, while unfermented tea alleviates inflammation (50). A randomized study has indicated that the consumption of both fermented and unfermented tea does not have a significant impact on reducing CRP and IL-6 levels (51). Furthermore, a systematic review of RCTs suggested that green tea (unfermented tea) may not be effective in altering the levels of CRP and IL-6, particularly in cases with low inflammation (52). A cross-sectional study including 3,042 participants showed that coffee consumption was related to an increase in inflammatory markers (53). An RCT also demonstrated that patients who consumed caffeinated coffee exhibited higher levels of inflammation compared to those who abstained from coffee consumption (54). Caffeine may play an important role in increasing the levels of inflammatory markers (55).

The study further revealed that dietary pattern with CRP as a response variable was associated with an increased risk of CI. Numerous studies have consistently demonstrated that a diet with pro-inflammatory properties is significantly associated with an elevated risk of CI (56–60). However, the finding is inconsistent with the results of the Nurses' Health Study, which discovered no correlation between a pro-inflammatory diet and cognitive function in a vast population primarily consisting of Caucasian and highly educated elderly women (61). Further research may be necessary to confirm the relationship between pro-inflammatory diets and cognitive function in men and other races. The consumption of tea and coffee was observed in the dietary pattern related to CI in the study. Noguchi-Shinohara's study has revealed that black tea intake is associated with an increasing risk of CI compared to green tea (62), indicating patients in the study may prefer the consumption of black tea. Catechin in green tea increased the expression of immediate-early genes in the hippocampus and prevented the decline of cognitive function (63). The consumption of green tea has also been shown to decrease Alzheimer's disease (AD)-related pathology and enhance anti-oxidative stress capacity, thereby achieving the objective of reducing CI risk (64). As mentioned before, the association between coffee consumption and cognitive function remains controversial. Some studies have suggested that coffee may improve cognitive decline in individuals with impaired cognition (65). Coffee has been shown to decrease lipid peroxidation in brain membranes, increase antioxidant levels, and enhance long-term memory (66). Additionally, coffee may reduce the risk of CI by decreasing pathological cerebral amyloid-beta (Aβ) deposition, which is a characteristic of AD (67). However, a meta-analysis including up to 415,530 participants did not find evidence for the effects of coffee consumption on global cognition or memory (68). In fact, higher intake of coffee was cross-sectionally associated with smaller hippocampal volume and poorer memory function (69). The association between coffee and CI in patients undergoing HD may depend on the amount of coffee consumed, and further investigation is warranted.

The “white rice” pattern, characterized by high consumption of white rice, flour-based food, and alcohol, was found to have a positive association with CI (70). Our result suggested that a high intake of white rice and liquor was associated with an increased risk of CI. The association between white rice and CI may be explained by low intake of micronutrients (71), as many studies have indicated associations between CI and antioxidant vitamins (vitamin E, vitamin C, and β-carotene) and B vitamins (vitamin B6 and folate) deficiency (72). The association between alcohol consumption and CI may be influenced by dosage. Long-term drinking has been linked to an increased risk of cognitive decline (73). A dose-response meta-analysis has demonstrated that low intake of alcohol intake (< 11 g/day) is associated with a reduced risk of CI (74), while individuals who abstain from drinking or consuming high levels of liquor have an elevated risk (75). Compared to non-drinkers, moderate lifetime alcohol consumption is associated with lower Aβ deposition (76). The findings in the study are consistent with previous research (77), which also suggested that the consumption of vegetables and juice is beneficial for cognition function. Similarly, our study found that dark vegetables and juice specifically have a positive impact on CI. Polyphenols found in vegetables and juice are associated with improved cognition (78). However, we observed an increased risk of CI associated with fruit consumption. Fruit with a high glycemic index or load may disrupt insulin signaling, impair glucose metabolism, and consequently result in neuronal loss, reduced cortical thickness, and CI (79).

The ultra-processed food (UPF) was not considered a distinct group in the study. However, three food groups, namely, desserts, processed meat products, and sweet beverages, belong to the UPFs. Weinstein and colleagues noted in a recent study that the consumption of ultra-processed meat, oils/spreads, and dairy products has been positively associated with cognitive decline in elderly individuals with type-2 diabetes, particularly among women and obese individuals (80). The UPFs are characterized by high levels of starches, fats, oils, sugars, emulsifiers, and food additives that have a direct link to CI (81). The underlying mechanisms for the impact of UPF on cognitive function may include low-grade chronic inflammation (82, 83). Desserts and processed meat products groups were not significantly correlated with CRP or IL-6 in the study. However, sweet beverages exhibited a positive correlation with IL-6 levels in patients undergoing HD, which potentially corroborates the relationship between the UPFs and CI. The UPF groups in the study failed to show a significant association with CI; this finding is inconsistent with previous research indicating that consumption of sugary beverages increases the risk of Alzheimer's disease (84). The inconsistency observed in the study may be attributed to the limited consumption of sweetened beverages reported by patients. Moreover, the link between UPFs and CI could be mediated through chronic conditions such as diabetes, cardiovascular disease, and obesity. Studies are needed to further examine the relationship between UPF and CI in patients undergoing HD.

## Strengths and limitations

Our study has several strengths. To the best of our knowledge, no previous studies have explored the association between dietary patterns, inflammatory biomarkers, and CI in patients undergoing HD. Our study was of great significance from the perspective of public health. A cost-effective strategy was given to patients undergoing HD to prevent the decline of cognitive function. However, several limitations should be considered. First, the causal relationship between dietary patterns derived from inflammation and CI was difficult to examine due to the cross-sectional study design. Second, dietary intake was obtained from FFQ, and there may be some recall bias. Finally, the dietary pattern derived from RRR explains the variation of response variables to the greatest extent, instead of the variation of food. A dietary pattern for dietary guidance should be obtained through factor analysis.

## Practical application

Encouraging and supervising more dark vegetables and low-sugar juice consumption may be one method of improving cognitive impairment by relieving inflammation in patients undergoing HD. However, the consumption of rice, tea, coffee, liquor, and high-sugar fruits should be prudent in order to alleviate inflammation and reduce cognitive decline. The intake of fruits and juice for patients undergoing HD may require the guidance of nephrologists and other healthcare professionals due to their sugar and potassium content. Additionally, the types of teas and coffees, as well as the method of cooking rice, may be contributing factors to inflammation reactions. Further exploration is necessary to clarify their relationship with cognitive function in patients undergoing HD. Meanwhile, strategies to improve inflammation control in individuals receiving HD should concentrate on the dietary restriction of seafood, sweet beverages, and liquor. However, the association between alcohol intake and inflammation requires further examination.

## Conclusion

This study provides patients undergoing HD with potential nonpharmacological, low cost dietary advice to improve cognitive function. The study suggests that dietary pattern, with CRP as a response variable, which is characterized by a high intake of rice, tea and coffee, liquor, and fruit and a low intake of dark vegetables and juice, is significantly associated with an increased risk of CI in patients undergoing HD. Consumption of seafood, sweet beverages, and liquor may be contributing factors to inflammation in patients; however, their association with CI has not yet been found.

## Data availability statement

The original contributions presented in the study are included in the article/supplementary material, further inquiries can be directed to the corresponding author.

## Ethics statement

This study was reviewed and approved by the Institutional Review Board at Nantong University (2021-48) and all participants provided informed consent prior to participation. The patients/participants provided their written informed consent to participate in this study.

## Author contributions

Conceptualization, methodology, writing—original draft preparation, and writing—review and editing: YZ, XW, and YS. Software: YZ. Validation: XW. Formal analysis: YZ and XW. Investigation, resources, and data curation: YZ, XW, XuZ, QF, and XiZ. Visualization, supervision, project administration, and funding acquisition: YS. All authors contributed important intellectual content during manuscript drafting or revision, accepts personal accountability for the author's own contributions, and agrees to ensure that questions pertaining to the accuracy or integrity of any portion of the study are appropriately investigated and resolved.
